# Can pyroptosis be a new target in rheumatoid arthritis treatment?

**DOI:** 10.3389/fimmu.2023.1155606

**Published:** 2023-06-22

**Authors:** Dengqiang Wu, Yujie Li, Ranxing Xu

**Affiliations:** ^1^ Department of Clinical Laboratory, Ningbo No.6 Hospital, Ningbo, China; ^2^ Department of Clinical Laboratory, Ningbo Medical Center Lihuili Hospital, Ningbo, China

**Keywords:** pyroptosis, rheumatoid arthritis treatment, inflammasome, gasdermin protein, caspase

## Abstract

Rheumatoid arthritis (RA) is a chronic systemic autoimmune disease of undefined etiology, with persistent synovial inflammation and destruction of articular cartilage and bone. Current clinical drugs for RA mainly include non-steroidal anti-inflammatory drugs (NSAIDs), glucocorticoids, disease modifying anti-rheumatic drugs (DMARDs) and so on, which can relieve patients’ joint symptoms. If we want to have a complete cure for RA, there are still some limitations of these drugs. Therefore, we need to explore new mechanisms of RA to prevent and treat RA radically. Pyroptosis is a newly discovered programmed cell death (PCD) in recent years, which is characterized by the appearance of holes in cell membranes, cell swelling and rupture, and the release of intracellular pro-inflammatory factors into the extracellular space, resulting in a strong inflammatory response. The nature of pyroptosis is pro-inflammatory, and whether it is participating in the development of RA has attracted a wide interest among scholars. This review describes the discovery and mechanism of pyroptosis, the main therapeutic strategies for RA, and the role of pyroptosis in the mechanism of RA development. From the perspective of pyroptosis, the study of new mechanisms of RA may provide a potential target for the treatment of RA and the development of new drugs in the clinics.

## Introduction

Rheumatoid arthritis (RA) is a chronic autoimmune disease characterized by symmetrical pain and swelling of multiple joints in the hands, wrists and feet. The pathology is characterized by persistent synovial inflammation, which can lead to articular cartilage erosion and joint deformity, is one of the main factors of disability (1–4). The global prevalence of RA is about 0.5-1.0% on average, and the quality of life of RA patients is lower than that of common diseases such as diabetes, myocardial infarction, hypertension and so on (5). Patients also suffer a huge financial burden of treatment.

Clinically available western medicines for RA include non-steroidal anti-inflammatory drugs (NSAIDs), glucocorticoids, disease-modifying anti-rheumatic drugs (DMARDs), biologics, botanicals, analgesics, etc. Most of these drugs can alleviate the symptoms of RA patients to a certain extent, but there are still many limitations such as adverse drug reactions and individual differences in drug efficacy (6–8). Therefore, the key to treat RA is to study the mechanism of RA and stop its development at the root. Recently, there is a large number of researches reporting that some Chinese medicines such as punicalagin, celastrol and BaiHu GuiZhi can treat RA through the pyroptosis pathway (9–12). Pyroptosis is a rapidly studied PCD in recent years, which is a form of cell death mediated by gasdermin protein, usually activated by cysteinyl aspartate specific proteinase (caspase), and accompanied with a strong inflammatory response (13–15). Morphologically, it appear holes in the plasma membrane, subsequently swelling with “light bulb” under the microscope (15–17). It has been demonstrated that chondrocytes, fibroblast-like synoviocytes (FLS), and monocytes undergo pyroptosis in RA patients (18). And high level of pyroptosis-related molecules such as NOD-like receptor thermal protein domain associated protein 3 (NLRP3), caspases, IL-18, IL-1β were detected in serum and synovial fluid (19, 20). All evidence suggests that pyroptosis is involved in regulating the pathogenesis of RA. Its role in RA has gradually attracted the attention of scholars (18, 21, 22). Therefore, this review describes the role of pyroptosis in the pathogenesis of RA and also provides new possible targets for the treatment of RA.

## Introduction of pyroptosis

### The origin of pyroptosis

Pyroptosis is a kind of PCD which has been studied deeply in recent years (13, 23, 24). It is an important non-specific defense mechanism in body, which plays a certain role in antagonizing the invasion of external pathogens and sensing endogenous danger signals (13, 23, 25, 26). In 1992, scholars such as Zychlinsky et al. observed that Shigellaflexneri could induce lytic death in infected host macrophages (27). This form of PCD was partly similar to apoptosis and was considered to be apoptosis at that time (27).

In 2001, Cookson BT et al. first described this mode of cell death as “pyroptosis”. “pyro” means “burning” in Greek and is used to indicate that the occurrence of such PCD accompanied by an inflammatory response, formally distinguishing it from apoptosis (28). The gasdermin (GSDM) family, core protein of pyroptosis was discovered in 2015 (16, 29). The study of pyroptosis has entered a whole new field since then. In 2018, the Nomenclature Committee on Cell Death (NCCD) recommended that the definition of pyroptosis be revised to a regulated cell death that depends on the GSDM protein family to form plasma membrane pores, which is often the result of inflammatory caspase activation (30).

### Mechanisms of pyroptosis

Gasdermin protein is the key of pyroptosis, including gasdermin A (GSDMA), gasdermin B (GSDMB), gasdermin C (GSDMC), gasdermin D (GSDMD), gasdermin E (GSDME, also known as DFNA5) and Pejvakin (PJVK, also known as DFNB59) (13). With the exception of PJVK, all other gasdermins consist of C-terminal domain (GSDM-CT) and N-terminal domain (GSDM-NT). In the resting state, GSDM-CT binds to GSDM-NT to inhibit the drilling activity of GSDM-N (31, 32). GSDM-NT will be released and freed to the inner surface of the cell membrane to bind to the phosphatidylinositol in the plasma membrane, forming a pore with an inner diameter of approximately 12-14 nm when the pyroptosis activation signal is triggered (29). Cell contents such as IL-18, IL-1 β and LDH are released to the outside of the cell through pores (16, 29, 33, 34). Due to the osmotic pressure inside and outside, the cells will swell with the fluid infiltration and eventually rupture and die. The activation pathway of pyroptosis can be divided into the canonical inflammasome pathway of caspase-1 activated by inflammasome and the noncanonical pathway of caspase-4/5/11 activated by lipopolysaccharide (LPS) (13, 29). GSDMD is the executive protein of pyroptosis, the common substrate of inflammatory caspase-1/4/5/11 enzymes, and the core of the two pathways (16, 29, 35, 36).

Inflammasome is a class of multi-protein complexes, the main types of which including NLRP1, NLRP3, NLRC4, AIM2 and so on (37–40). The basic structure of inflammasome includes three parts: pattern recognition receptor (PRR), apoptosis-associated speck-like protein containing CARD (ASC) and pro-caspase-1 (41, 42). PRRs recognize dangerous signals. Different inflammasome will be activated by a variety of corresponding activation ways when they are stimulated by different external signals. ASC will recruit PRRs and pro-caspase-1 through the oligomerization of pyrin domain (PYD) and C-terminal caspase recruitment domain (CARD) (41, 43, 44), then assemble inflammasome complexes for downstream signal transduction. As an effector molecule, pro-caspase-1 is recruited by ASC to form activated caspase-1 (41, 45, 46).

**Figure 1:**
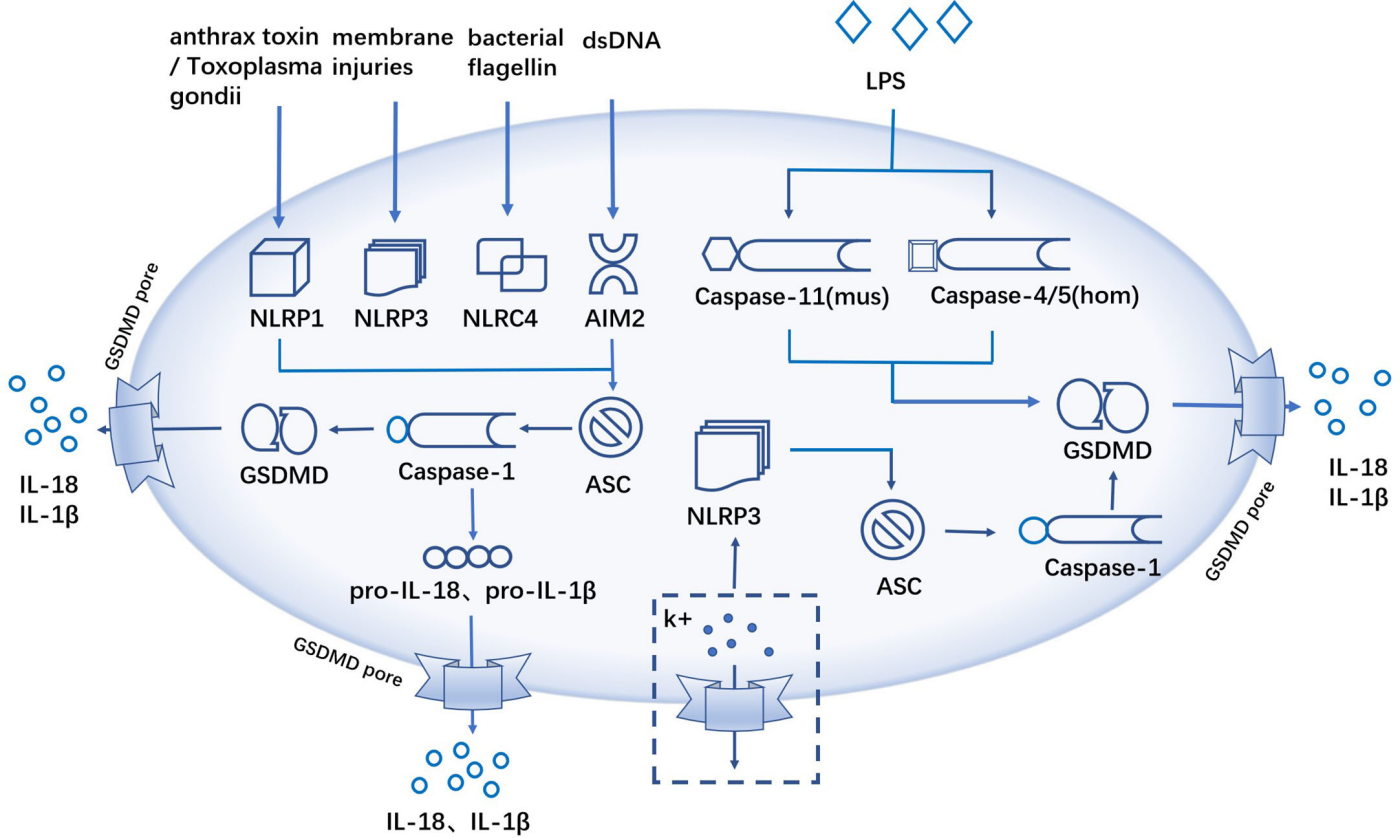
The canonical and noncanonical inflammasome pathway. The canonical inflammasome pathway, anthrax toxin/Toxoplasma gondii infections, membrane injuries, bacterial flagellin and dsDNA activate inflammatory and ASC recruits pro-caspase-1 to form inflammatory complexes, activated caspase-1 cleaves GSDMD, and the cellular contents like IL-18, IL-1β and LDH are released extracellularly through the GSDMD pores. The non-canonical inflammasome pathway, caspase-11 (mouse) or caspase-4/5 (human) sensing LPS, promotes pyroptosis via GSDMD cleavage. K+ efflux activates NLRP3 inflammatory and activated caspase-1 induces pyroptosis.

In the canonical inflammasome pathway, NLRP1 is activated by anthrax toxin and Toxoplasma gondii infection (47); NLRP3 is activated by various membrane injuries; NLRC4 inflammasome recognizes bacterial flagellin (48, 49); AIM2 acts as a DNA-binding receptor to recognize double-stranded DNA (dsDNA) (40). Then ASC recruited pro-caspase-1 to develop inflammasome, and the activated caspase-1 further cleave the downstream GSDMD protein to promote the generation of pyroptosis (16, 36, 46). Different from the canonical pathway of pyroptosis, caspase-11 (mouse) or caspase-4/5 (human) can start the activation process by sensing the expression of LPS directly, which form pores in the cell membrane through GSDMD cleavage and promote GSDMD-mediated pyroptosis, a noncanonical pathway of pyroptosis (29). In addition, the researchers found that K+ efflux can activate NLRP3 inflammasome (50), which further induce caspase-1-mediated pyroptosis (50, 51) (**Figure 1**).

In addition to the canonical and noncanonical pyroptosis pathway, there are other activation types of gasdermins-mediated pyroptosis. Chemotherapeutic drugs such as paclitaxel and cisplatin can activate pro-caspase-3, which in turn mediates pyroptosis by cleaving GSDME (15). This study also showed that more “apoptosis” can be transformed into “pyroptosis” in the regulation of GSDME (15, 52). Granzyme B is a serine protease that is mainly expressed by NK cells or cytotoxic T lymphocytes (53, 54). Granzyme B can cut GSDME at the same site like caspase-3 and induce *GSDME*-mediated pyroptosis (55). A study on melanoma cells found that iron significantly increased chemotherapy-triggered reactive oxygen species (ROS), promoting the oxidation and oligomerization of the outer mitochondrial membrane protein Tom20. Activated Tom20 recruited Bax to the mitochondria, which then induced further activation of caspase-3 by cytochrome C and ultimately trigger *GSDME*-mediated pyroptosis in melanoma cells (56). Granzyme A can enter target cells via perforin and induce pyroptosis by cleaving GSDMB protein at the Lys244 site. And GSDMB protein is currently recognized as an executor protein that can be cleaved by Granzyme A (57). In hypoxic environment, p-STAT3 interacts with PD-L1 physically to enhance the transcription of *GSDMC*. At the same time, under the treatment of TNF-α, activated caspase-8 cleaves GSDMC in MDA-MB-231 breast cancer cells to induce *GSDMC*-mediated pyroptosis (58).

## Application of Western and Chinese medicines in RA treatment

Rheumatoid arthritis is a chronic systemic autoimmune disease, and its pathogenesis is not clear yet (59, 60). The main clinical manifestations are symmetrical and invasive inflammation of small joints such as hands and feet, which can lead to joint deformities and function loss, and even involve heart and lung (5, 61, 62). From a pathological point of view, RA is a widespread inflammatory disease that mainly involves articular synovium, which can spread to articular cartilage, bone tissue and joint ligament (62–64). Western medicines and Chinese herbs are available for the treatment of RA.

At present, western medical treatment is mainly based on NSAIDs, disease-modifying antirheumatic drugs (DMARDs) and glucocorticoids (65–68). The main mechanism of NSAIDs is to inhibit cyclooxygenase (COX) to prevent the synthesis of inflammatory mediators such as prostaglandins and thromboxane, thus exerting anti-inflammatory and analgesic effects. Representative drugs include meloxicam tablets, diclofenac, aspirin and so on (69–71). Considerable progress has been made in the development of DMARDs, which are effective in preventing joint imaging damage in patients with RA. This kind of drugs include conventional synthetic disease modifying anti-rheumatic drugs(csDMARDs), biological disease modifying anti-rheumatic drugs(bDMARDs) and target synthetic disease modifying anti-rheumatic drugs(tsDMARDs) (66, 72, 73). The commonly used csDMARDs include methotrexate (MTX), sulfasalazine (SASP), leflunomide (LEF), penicillamine and so on. MTX is generally the first choice for the treatment of RA, mainly through the inhibition of dihydrofolate reductase, reducing the production of inflammatory factors to achieve immunosuppressive effects (74–76). SASP can reduce the level of immunoglobulin, reduce the secretion of IL-8 and monocyte chemoattractant protein, thereby interfering with T-cell function (77). LET is a new type of antimetabolic immunosuppressant, whose mechanism is to inhibit dihydroorotic acid dehydrogenase and tyrosine kinase activity and to attenuate the activation of lymphocyte growth (78, 79). Tofacitab and baricitinib are tsDMARDs, which interfere with the production of downstream inflammatory cytokines by inhibiting Janus kinase (JAK), thereby preventing the further development of RA (80–82). The main purpose of bDMARDs is to inhibit various core inflammatory factors of RA to achieve the purpose of treating and alleviating joint injury. Etanercept is a common TNF-α inhibitor that competitively binds to TNF-α and exerting anti-inflammatory effects (83). Anakinra, a human-derived IL-1 receptor antagonist, is greatly limited in the treatment of RA due to its short half-life (84). Sarilumab is a humanized monoclonal antibody that inhibits IL-6-mediated signal transduction and mitigates the progression of RA (85, 86). Rituximab is a human-mouse chimeric anti-CD20 monoclonal antibody, which can significantly reducing the secretion of autoantibodies and rheumatoid factors in the body (87, 88). The above-mentioned bDMARDs drugs are targeted and effective in relieving RA symptoms, but also have the disadvantage of increasing the risk of infection (89, 90). Glucocorticoids have powerful anti-inflammatory effects and are mainly used clinically in small doses and short courses for the treatment of RA, providing rapid relief of joint swelling and inflammation (91–93). The main clinically applied glucocorticoids are hydrocortisone, dexamethasone and prednisone. However, glucocorticoid cannot completely treat RA and has serious side effects such as atherosclerosis, hypertension, infection, osteoporosis and so on (89, 90).

Chinese medicine has a good efficacy in treating RA (94). Recent studies have found that some Chinese medicines can be mediated through pyroptosis pathway in the prevention and treatment of RA. Therefore, it may provide some new targets for clinical drug development of RA if scholars can study the mechanism of Chinese medicine against RA from the perspective of pyroptosis.

Ling et al. found that Hmong Jin Wu Jian Wu Bone Capsules were able to inhibit fibroblast-like synoviocyte (FLS) pyroptosis through the *NLRP3/caspase/GSDMD* pathway in the treatment of RA (95). Ge et al. found that punicalagin was able to reduce the expression of NLRP3 inflammatory in collagen-induced arthritis (CIA) mice and inhibit the occurrence of pyroptosis, resulting in an anti-inflammatory effect (9). Celastrol is a quinone methylated triterpenoid that is used in the treatment of RA. Studies have shown that celastrol can block the nuclear factor kappa-B (*NF-κB*) signaling pathway, inhibiting the activation of NLRP3 inflammatory. In addition, it also inhibits LPS and adenosine triphosphate (ATP)-induced ROS. Therefore, celastrol may play a role in the treatment of RA by inhibiting the *ROS/NF-κB/NLRP3* signaling pathway (10). The Chinese medicine BaiHu GuiZhi can attenuate the inflammatory response of RA by inhibiting the activation of Toll-like receptor 4(*TLR4*)-mediated NLRP3 inflammasome. The increased levels of NLRP3, caspase-1, LDH, IL-1β and IL-18 were significantly reversed by Baihu Gui Zhi, confirming the immunomodulatory and anti-inflammatory activities of Baihu GuiZhi especially in pyroptosis (11, 12). Cinnamaldehyde, an important component of cinnamon, inhibits the expression of *NLRP3* and *HIF-1α* to reduce the level of IL-1β, thereby alleviating the synovial inflammatory response in adjuvant arthritis (AA) rats (96). Paeoniflorin was extracted from Paeonia lactiflora and its monomeric derivatives were found to exert therapeutic effects in rats with AA by regulating *TLR4/NLRP3/GSDMD* pathway and inhibiting macrophage pyroptosis (97). Chinese herb Taraxasterol can inhibit FLS pyroptosis by regulating the NLRP3 inflammatory to play an anti-RA role (98).

## Some key cells promote RA progression through pyroptosis

### Fibroblast-like synoviocyte, FLS

**Figure 2:**
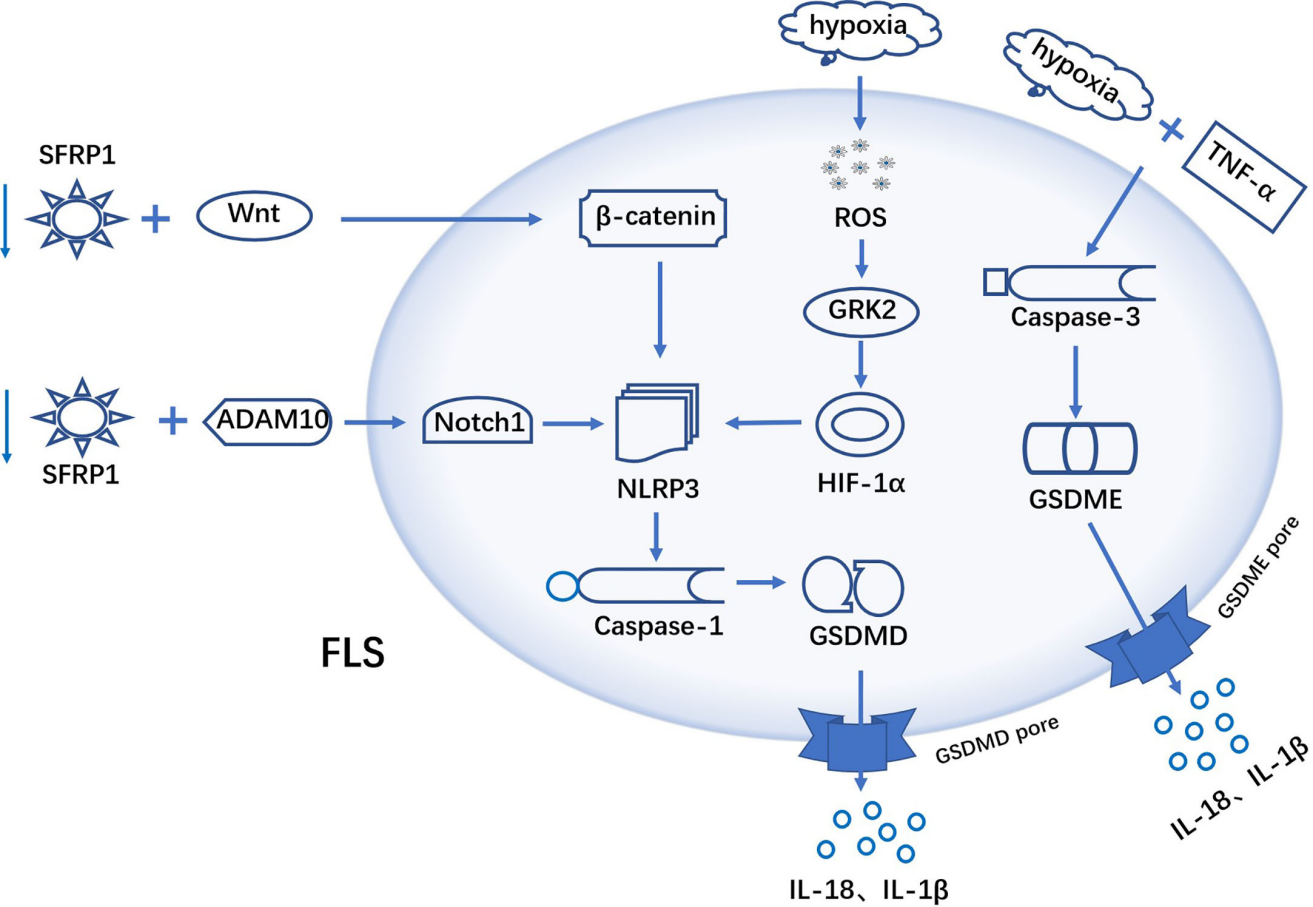
Mechanism of FLS pyroptosis in RA TNF-α plus hypoxia induces GSDME-mediated pyroptosis in FLS. ROS in FLS were elevated under hypoxic conditions. Excess ROS promoted *GRK2* expression, which in turn increased the synthesis of HIF-1α. HIF-1α activates the transcription of *NLRP3* inflammasome, excess NLRP3 activate caspase-1, and activated caspase-1 cuts GSDMD. b-catenin interacts with NLRP3 to activate NLRP3 inflammasome and initiate pyroptosis. SFRP1 binds to ADAM10 metalloprotein and down-regulates its activity, leading to blocking the activation of Notch signaling. A hypothesis: SFRP1 negatively regulates *NLRP3*-mediated pyroptosis via *wnt/b-catenin* and *Notch* signaling pathways in RA-FLS.

Fibroblast-like synoviocyte (FLS) is the main cell forming the synovial structure and has an important role in the inflammatory response to RA and joint damage. Abnormally activated FLS exhibits “tumor-like” properties and is in an activated proliferative state. Overproliferation and insufficient apoptosis of FLS play a key role in the destruction and persistent inflammation of RA (99, 100). FLS in RA patients can cause synovial proliferation, inflammatory response and bone destruction by producing large amounts of pro-inflammatory cytokines, matrix metalloproteinases (MMPs) (101). Abnormally overproliferating FLS migrates to unaffected joints, attaches to bone tissue and resists apoptosis (102, 103). Therefore the proliferation of FLS contributes to the development of RA.

pyroptosis is a PCD accompanied by inflammatory response, and recent studies have found that pyroptosis in FLS promote the progression of RA. It has been shown that increased GSDME expression in RA synovial tissue and tumor necrosis factor-α (TNF-α) plus hypoxia induce GSDME-mediated pyroptosis in FLS, with a concomitant increase of migration and invasion (104). This study links aberrantly activated FLS to the inflammatory and hypoxic synovial microenvironment and provides a new idea for fls-targeted therapy in RA. Another research, under hypoxic conditions, ROS levels within FLS increased dramatically. Excess ROS promotes the expression of GRK2, which increases the synthesis of HIF-1α. HIF-1α is translocated to the nucleus and initiates transcription of NLRP3 inflammasomes. High levels of NLRP3 inflammasomes, activate caspase-1, which in turn cuts the GSDMD thereby triggering FLS pyroptosis (105) (**Figure 2**).

β-catenin interacts with NLRP3 and promotes its conjugation to ASC, which activates NLRP3 inflammatory and triggers pyroptosis (106). Notch1 is a novel factor that mediates the activation of NLRP3 inflammasomes. Secreted Frizzled-Related Protein 1(SFRP1) can bind to ADAM10 metalloprotein and down-regulate its activity, thereby inhibiting the activation of Notch signaling (107, 108). In the study by Jiang et al, they hypothesized that in RA-FLS, SFRP1 can negatively regulate *NLRP3*-mediated pyroptosis through *Wnt/b-catenin* and *Notch* signaling pathways (109). Therefore, this implies that blocking the activation of downstream signaling by upregulating the expression of *SFRP1* in RA-FLS would contribute to the treatment of RA (**Figure 2**). In addition, Yang et al. have shown that LPS could induce FLS pyroptosis through two signaling pathways, *NLRP-3/caspase-1/GSDMD* and *caspase-3/GSDME*. NF-κB, as an upstream molecule of these two signaling pathways, is also involved in the activation of FLS pyroptosis (110).

Circular RNAs (CircRNAs) is a class of non-coding RNA molecules that do not have a 5’ end cap and a 3’ end poly(A) and are covalently bonded to form a circular structure. Hsa_circ_0044235 is expressed at lower levels in RA patients than in normal (111). Overexpression of Hsa_circ_0044235 inhibits *NLRP3*-mediated FLS pyroptosis, and this regulation may be achieved through the miR-135b-5p-SIRT1 signaling axis (112).

## Monocytes/macrophages

**Figure 3:**
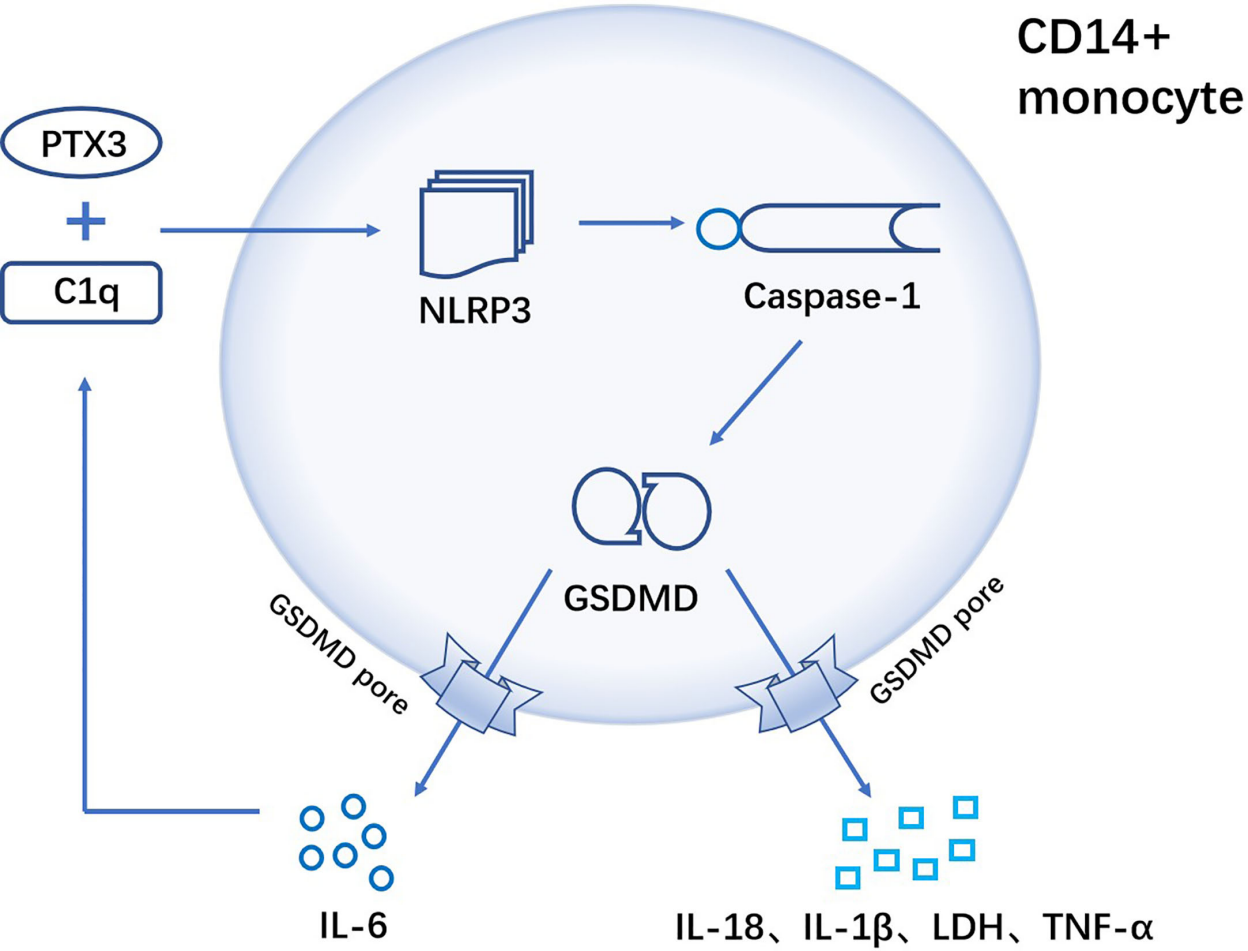
C1q and PTX3 act synergistically to induce GSDMD-dependent pyroptosis of CD14+ monocytes in RA. In CD14+ monocytes, the combination of C1q and PTX3 induces NLRP3 inflammasome, followed by the activation of caspase-1. Activated caspase-1 further cleaves the pyroptosis execution protein GSDMD, and GSDMD-N frees to the cell membrane to form pores that result in pyroptosis. Cellular contents are released from the GSDMD pore (TNF-α, IL-1β, IL-18, IL-6 and LDH), where IL-6 can positively enhance C1q+PTX3 combination to mediate GSDMD-dependent pyroptosis.

Pentraxin 3(PTX3), an important component of innate immunity (113), is elevated in the serum of RA patients and is considered as a new marker for the diagnosis of rheumatoid arthritis (114). In RA patients, complement 1q (C1q) and PTX3 act synergistically to induce GSDMD-dependent pyroptosis in CD14+ monocytes, while promoting the release of inflammatory cytokines (tumor necrosis factor-α, IL-1β and IL-6). And the released IL-6 further promotes PTX3 plus C1q-induced pyroptosis in normal monocytes (21) (**Figure 3**).

**Figure 4:**
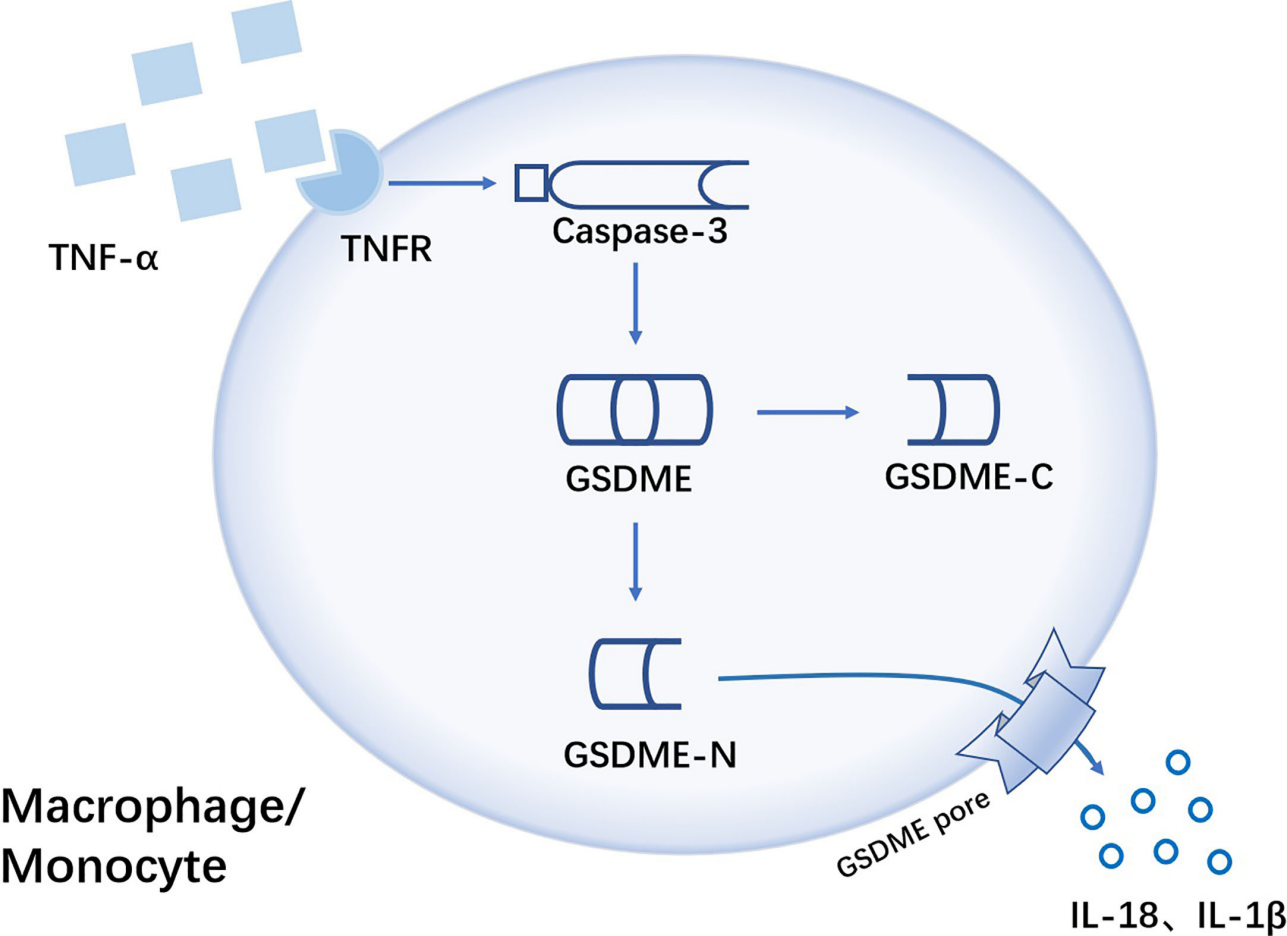
TNF-α induces GSDME-mediated pyroptosis of monocytes/macrophages in RA TNF-α is sensed by Tumor necrosis factor-α receptor(TNFR) on the surface of monocytes/macrophages, with the activation of caspase-3. Then activated caspase-3 cleaves GSDME, followed by free GSDME-N playing a pore-forming role leading to GSDME-dependent pyroptosis. Pro-inflammatory cytokines like IL-18 and IL-1β are released from GSDME pore.

The expression of pyroptosis-related proteins caspase3 and GSDME-N were increased in monocytes and macrophages of RA patients. And by activated caspase3-GSDME signaling pathway, tumor necrosis factor-α (TNF-α) could induce pyroptosis of monocytes and macrophages (**Figure 4**). In CIA mice, GSDME deficiency effectively attenuated the degree of joint damage (22), so targeted inhibition of GSDME may be a potential treatment for RA.

DNA polymerase β (POL β), which is a key enzyme for base excision repair (115), has been shown to play a critical role in the pathogenesis of RA. POL β was lowly expressed in peripheral blood mononuclear cells (PBMC) of RA patients and CIA mice. Pol β deficiency promoted LPS plus ATP-induced pyroptosis in macrophage. And polβ knockdown promoted pyroptosis by activating the *cGAS-STING-NF-κB* signaling pathway, which upregulated NLRP3, IL-1β and IL-18 (116). As a new mechanism of macrophage pyroptosis in RA patients, it provides a new direction for the development of anti-RA drugs and treatment.

## Chondrocyte

Acid-sensitive ion channels(ASICs) is a class of extracellular proton-activated cation channels whose main role is to permeabilize Na+ and Ca2+ (117). There are 7 subunits: ASIC1a, ASIC1b, ASIC1b2, ASIC2a, ASIC2b, ASIC3, ASIC4, of which ASIC1a is the only subunit that transports Ca2+, while the others are all Na+ permeable channels (118, 119). Acid-base balance is one of the most significant requirements for the maintenance of normal physiological activity (120). Almost all diseases, such as inflammation, hypoxia, cancer, etc., can cause pH changes to different degrees. Changes in pH can be sensed by the body through ASICs, which in turn regulate the corresponding tissue changes (121, 122).

The main pathological feature of RA is persistent synovitis, which leads to the destruction of articular cartilage and bone, and the accumulation of inflammatory metabolites in the joints of RA patients leads to localized tissue acidification. Acidified joint fluid can lead to apoptosis of chondrocytes, which can exacerbate the symptoms of RA (123). Acidification of joint fluid can significantly induce apoptosis in articular chondrocytes, and Wu et al. found that pyroptosis is also involved in the pathological changes. They found that extracellular acidosis significantly increased the expression of pyroptosis related protein such as ASIC1a, ASC, NLRP3 and caspase-1 in chondrocytes, while promoting the release of LDH, IL-1b and IL-18. The expression of pyroptosis associated molecules was decreased with the ASIC1a inhibitors amiloride and Psalmotoxin-1 (Pctx-1) (124). These results suggest that ASIC1a may trigger chondrocyte pyroptosis in adjuvant arthritis (AA) rat by elevating [Ca2+]i.

The inhibition of ASICs may provide a new idea for the treatment of RA, but the drugs amiloride has been found to be non-selective for ASICs, while the animal toxin represented by Pctx1 is selective but with high toxicity (125, 126), so finding a suitable ASICs inhibitor will determine whether the ASICs therapy can be applied clinically.

MicroRNAs play a very important role in the regulation of gene expression as non-coding single-stranded RNAs (127). A number of studies have shown an association between microRNAs and the development of RA (128–131). The expression of NLRP3, GSDMD, cleaved caspase-1 and cleaved caspase-3 in N1511, a mouse chondrocyte, was upregulated after IL-1β stimulation. Pyroptosis was inhibited by downregulation of miR-144-3p in N1511 cells, and miR-144-3p-activated chondrocyte pyroptosis was regulated by the PTEN/PINK1/Parkin signaling axis (132).

## CD4+ T cell

**Figure 5:**
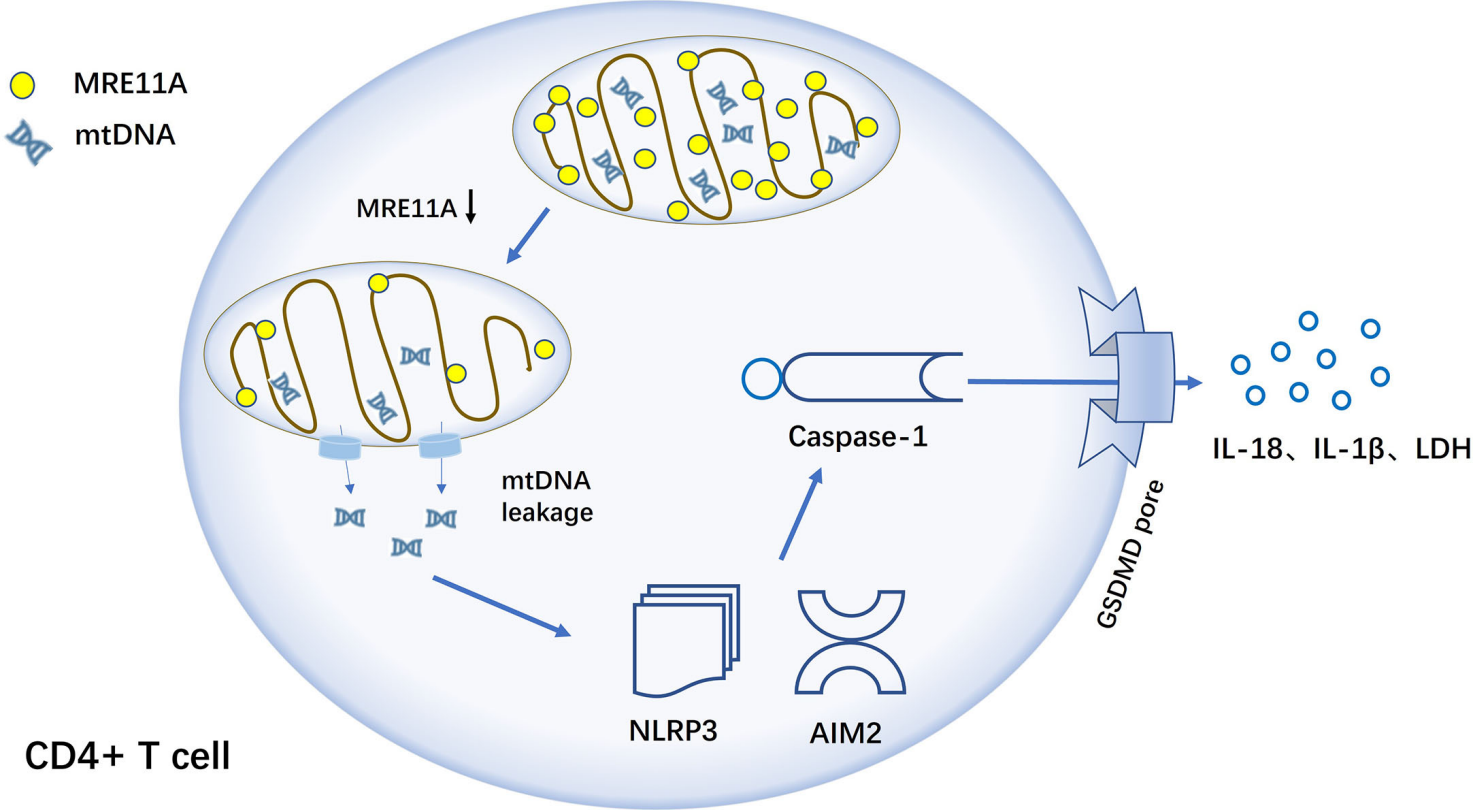
MRE11A loss of function results in caspase-1-induced pyroptosis of CD4+ T cell in RA T cells in RA lack MRE11A and mtDNA leak into the cytoplasm, activating inflammasome NLRP3 and AIM2, followed by further caspase-1 activation, triggering caspase-1-mediated pyroptosis and causing tissue inflammation.

MRE11A is an enzyme that helps to repair single and double-stranded nucleic acid breaks (133–135). Decreased MRE11A expression of CD4+ T cells in RA patients results in diminished mitochondrial capacity (136) and release of mtDNA (mitochondrial DNA) into the cytoplasm and triggers inflammation. In a humanized NSG mouse model, MRE11A low T cells exhibit tissue invasiveness and strong pro-inflammatory properties, while recombinant MRE11A alleviates the symptoms of tissue synovitis (137). Mechanistic studies show that in MRE11A low T cells, oxidized mtDNA leaks into the cytoplasm, triggering NLRP3 and AIM2 inflammasomes assembly (138), with caspase-1 activation, leading to GSDM-dependent pyroptosis (**Figure 5**).

In RA, T cells are converted into effector cells with an inflammatory response that promotes the progression of the disease. Therefore, up-regulating the expression of MRE11A in RA CD4+ T cells to reduce caspase-1 activation and the generation of pyroptosis, which can achieve the effect of slowing down the progression of synovitis in patients (133). MRE11A, as a repair enzyme for mtDNA, may play a key central role in the development of RA and is one more potential target for the clinical treatment of RA. A new, reliable and effective RA drug can be developed by blocking a link in the pyroptosis activation pathway from the perspective of CD4+ T cell death form, prolonging T cell lifespan and avoiding T cell-mediated inflammatory responses.

## Progress in the study of pyroptosis key protein inhibitors

Inhibitors of NLRP3, a key protein in the pyroptosis pathway, such as MCC950, OLT1177, CY-09, and the FDA-approved drug disulfiram (GSDMD inhibitor), we summarize the current mechanism of these drugs and their exploration in the clinical trials (**Table 1**). The discovery of these drugs provides a reference for their future clinical application against inflammatory diseases caused by pyroptosis.

**Table 1:** Research progress of pyroptosis key protein inhibitors.

Inhibitor	Target	Research progress	Refs
**MCC950**	NLRP3	MCC950 can inhibit the activation of NLRP3 by preventing the hydrolysis of ATP to ADP through non-covalent binding to Walker B in the NLRP3 NACHT domain. MCC950 has been used in a wide range of diseases involving NLRP3, including cryopyrin associated periodic syndrome (CAPS), nonalcoholic steatohepatitis (NASH), rheumatism, Alzheimer’s disease and so on. However, it has been discontinued in clinical phase II due to hepatotoxicity.	(139–141)
**miRNA-20a**	NLRP3	MiRNA-20a targets TXNIP to inhibit the activation of NLRP3, ASC and caspase-1 in adjuvant-induced arthritis FLS and to suppress the secretion of IL-1 and MMP-1.	(142)
**miRNA let-7f-5p**	NLRP3	let-7f-5p was shown to be a new promising treatment for SLE, which alleviated inflammation in SLE-BMSCs by targeting NLRP3 directly.	(143)
**OLT1177**	NLRP3	OLT1177 targets NLRP3 to reduce the catalytic activity of the ATP binding site directly and blocks the recruitment of NLRP3 to ASC, is currently in clinical Phase II, which is safe in humans.	(144, 145)
**CY-09**	NLRP3	CY-09 directly binds to the ATP-binding region of NLRP3, inhibiting its ATP hydrolytic activity and blocking inflammasome assemble subsequently. Moreover, CY-09 showed good efficacy in mouse models of CAPS and type 2 diabetes mellitus.	(146, 147)
**Oridonin**	NLRP3	Oridonin inhibits the activation of NLRP3 by covalently binding to C279 of NACHT in NLRP3.	(148, 149)
**INF39/INF58**	NLRP3	Through covalent binding of NLRP3 and affecting its function of hydrolyzing ATP, INF39 blocks its activation.	(150, 151)
**Tranilast**	NLRP3	Tranilast binds directly to the NACHT structural domain of NLRP3 and inhibits the assembly of NLRP3 inflammasome, showing therapeutic effects in gouty arthritis and type 2 diabetes mellitus mouse models.	(152–154)
**Disulfiram**	GSDMD	Metabolites of disulfiram undergo covalent bonding at cysteine position 191 of GSDMD and inhibit the pore-formation of GSDMD. Survival of mouse sepsis model activated by LPS-induced pyroptosis was significantly increased under disulfiram treatment.	(155)
**necrosulfonamide (NSA)**	GSDMD	NSA disrupts the GSDMD-N-terminal oligomerization and pore formation by alkylating the cysteine 191 position in the GSDMD.	(156, 157)
**Dimethyl fumarate (DMF)**	GSDMD/GSDME	DMF modification at Cys191 of GSDMD and Cys45 of GSDME to form S-(2-succinyl)-cysteine. Succinylation of GSDMD and GSDME stopped the induction of pyroptosis. In mouse models, the delivery of DMF protected them from LPS shock as well as attenuated familial Mediterranean fever (FMF) and experimental autoimmune encephalitis (EAE) by targeting GSDMD.	(158)

## Other PCDs participate in the pathogenesis of RA

PCD has been extensively studied and identified as one of the essential pathological mechanisms of RA, and there is a close association between multiple types of PCD and RA. In addition to pyroptosis, other forms of PCD such as ferroptosis, apoptosis, necroptosis and autophagy have been shown to be involved in the pathogenesis of RA (18, 160, 161). Therefore, targeting PCD therapy could be a potential therapeutic strategy.

FLS exhibits resistance to apoptosis during the progression of RA, leading to synovial proliferation and promoting the release of associated cytokines through diverse mechanisms (162, 163). RA-FLS is characterized by tumor-like behaviors such as activation, invasion and migration, as well as promoting the destruction of bone and cartilage, so apoptosis-induction in RA-FLS is an effective strategy for the treatment of RA (164). Bcl-2 and Mcl-2 anti-apoptosis proteins are up-regulated in the FLS of RA patients, indicating that apoptosis is inhibited in the development of RA, and how to effectively promote FLS apoptosis and inhibit synovial proliferation has important clinical implications for RA treatment (165). Necroptosis is a mode of PCD distinct from apoptosis and necrosis. Similar to apoptosis, it strictly follows the regulation of intracellular signaling pathways and possesses the morphological characteristics of necrosis (166–168). As a non-caspase-dependent mode PCD, it can be triggered by the binding of death receptors such as TNFR or Toll-like receptors to ligands in the absence of intracellular caspase signaling factors. And the mechanism is related to the cascade activation of receptor-interacting protein kinase 1(RIPK1), receptor-interacting protein kinase 3 (RIPK3) and the downstream mixed lineage kinase domain-like protein (MLKL) (167, 169–171). Neutrophils in the joints of RA patients undergo necroptosis with upregulated expression of RIPK1, RIPK3 and MLKL (172). Studies in the articular cartilage of AA rats have shown increased expression of RIPK1, RIPK3 and P-MLKL mediated by ASIC-1a. The use of Necrostatin-1, an inhibitor of RIPK1, reduced joint damage in AA rats (173).

Ferroptosis is an iron-dependent PCD accompanied by iron accumulation and lipid peroxidation (160, 174). ROS, GPX4 and iron accumulation have been found to be closely associated with the development of RA in recent studies, indicating a likely link between RA and ferroptosis-related pathological processes (175). In the development of RA, FLS produces the pro-inflammatory cytokine TNF-α, which leads to synovitis and bone destruction (176, 177). As a product of oxidative stress, ROS are present in large quantities in the joint lumen of RA and pathological ROS imbalance affects the progression of RA through ferroptosis (178, 179). GPX4, a core molecule of ferroptosis, can effectively inhibit lipid peroxidation and block ferroptosis in cells (180, 181). In several drug studies for RA treatment, a decrease in inflammatory factors, mainly TNF-α, was observed in conjunction with an increase in GPX4 expression (161, 175). Drugs targeting ferroptosis could be a potential strategy for RA treatment.

Autophagy is a non-apoptosis form of PCD, which ensures the maintenance of cellular homeostasis by removing damaged organelles and waste proteins through the autophagosome-lysosome pathway (182, 183). Patient with RA have a significant increase in the expression of autophagy-related proteins such as Beclin1, ATG5 and LC3 in synovial tissue (184). By expressing different molecular patterns, FLS enhances autophagy and anti-apoptosis to promote its proliferation. In addition, the development of autophagy by T cells, osteoblasts and osteoclasts has been connected to RA (163, 185–187). Thus, the phenomenon of autophagy is strongly associated with the progression of RA.

The various PCD do not exist in isolation in the normal organism. Though the signaling pathways between pyroptosis, apoptosis, necroptosis and ferroptosis are different, they are not unrelated and these different PCD pathways are closely linked and regulated by each other (18, 160, 168). For example, ROS plays a key role in regulating iron overload-induced necroptosis in osteoblasts, while RIPK1/RIPK3/MLKL is a key pathway in iron-induced necroptosis (188). Iron-mediated ferroptosis can be involved in the induction of pyroptosis. The combination of iron and ROS-inducing drugs can cause a significant increase of ROS levels in melanoma cells, leading to pyroptosis via the ROS-Tom20-Caspase 3-GSDME signaling pathway (56). The study by wang et al. found that the presence of GSDME promotes a shift from TNFα-induced apoptosis to pyroptosis (15), and though this study was conducted on tumor cells, RA-associated cells may have similar mechanistic links and changes. Several PCDs involved in the pathogenesis of RA cooperate with each other and synergistically contribute to the multiple pathological processes of RA. Several PCDs involved in the pathogenesis of rheumatoid arthritis cooperate with each other and contribute to the multiple pathological processes of rheumatoid arthritis. Further study on the role of these PCDs in RA will have profound implications for the early diagnosis, treatment and prognosis of RA.

## Conclusion

RA is a multisystemic inflammatory autoimmune disease involving peripheral joints, and the pathogenesis is still unclear. While greater progress has been made in the treatment of RA in recent years, such as the clinical application of some DMARDs, the pathogenesis of RA remains to be further explored. Both canonical and noncanonical activation pathways of pyroptosis as a pro-inflammatory death modality can induce strong inflammatory responses in body. Scientists have found in the results of numerous experimental studies that the progression of RA is closely associated with pyroptosis. IL-18 and IL-1β are pro-inflammatory cytokines released by cells undergoing pyroptosis, and they both belong to the interleukin-1 family, which has biologically similar inflammatory functions. IL-1β, as a product after inflammatory vesicle activation, promotes the binding of receptor activator of NF-kB (RANK) to ligands and promotes overactivation of RA-osteoclasts (189). *IL-18* is secreted at significantly higher level in the synovial fluid and serum of RA patients than in normal, where it acts as an angiogenic mediator and leukocyte chemotactic agent, mediating the inflammatory process in RA (190–193). Knockdown of *IL-18* gene resulted in reduced arthritic symptoms and decreased pro-inflammatory factors in RA mouse models, and inhibitors of caspase-1 reduced serum concentrations of IL-18 and IL-1β in CIA mice (194). All signs indicate the involvement of pyroptosis in the progression of RA. Absent in melanoma 2 (AIM2) is a member of pyroptosis and PANoptosis pathway. The latest studies have found that it is aberrantly expressed in a variety of immune cells such as T cells, B cells, FLS, etc. and that it mediates RA through a related molecular mechanism. There are several targeted inhibitors of AIM2, but their effectiveness in treating RA is unknown (195). Certain Chinese medicines, such as punicalagin, Celastrol, BaiHu GuiZhi, Cinnamaldehyde, etc., have obvious effects in anti-RA process, and mechanistic studies have revealed that their prevention and treatment of RA is through the pyroptosis pathway. There is also abundant evidence showing that chondrocytes, FLS, monocytes and CD4+ T cells in the RA organism undergo pyroptosis (**Table 2**). Therefore, inhibition of cell pyroptosis is very important for the treatment of RA, which can be used as a new drug target to control RA by blocking a certain part of pyroptosis. In addition to pyroptosis, a number of other PCDs including necroptosis, autophagy and ferroptosis are also involved in the pathogenesis and molecular regulation of RA. And these PCDs are connected and interact with each other, but the clear mechanisms and pathways involved are not fully clarified.

**Table 2:** Mechanism of the key cells in RA development.

Cells	Different types of cells undergo pyroptosis	Refs
**FLS**	1、TNF-α plus hypoxia induce GSDME-mediated pyroptosis in FLS. 2、Hypoxia trigger GSDMD-mediated pyroptosis through ROS-GRK2-HIF-1α-NLRP3-caspase-1-GSDMD signal axis. 3、SFRP1 can negatively regulate NLRP3-mediated pyroptosis through Wnt/b-catenin and Notch signaling pathways in RA-FLS. 4、LPS can induce FLS pyroptosis through NLRP-3-caspase-1-GSDMD and caspase-3-GSDME signaling pathways. 5、Hsa_circ_0044235 inhibit NLRP3-mediated FLS pyroptosis through the miR-135b-5p-SIRT1 signaling axis.	(104–106, 108, 110, 112)
**Monocytes/** **Macrophages**	1、C1q and PTX3 act synergistically to induce GSDMD-dependent pyroptosis in CD14+ monocytes, and the released IL-6 can further promotes pyroptosis in normal and RA monocytes. 2、TNF-α can induce pyroptosis through caspase-3-GSDME pathway in RA-monocytes/macrophages. 3、Pol β deficiency promote LPS plus ATP-mediated pyroptosis in RA-macrophage by activating the cGAS-STING-NF-κB signaling pathway. 4、MiR-33 promotes RA-macrophage pyroptosis by positively regulating NLRP3.	(21, 22, 116, 159)
**Chondrocyte**	1、Extracellular acidosis trigger chondrocyte pyroptosis and pyroptosis related protein such as ASC, NLRP3 and caspase-1 are significantly increased in RA-chondrocyte. 2、MiR-144-3p-activated chondrocyte pyroptosis is regulated by the PTEN/PINK1/Parkin signaling axis.	(124, 132)
**CD4+ T cell**	1、In MRE11A ^low^ CD4+ T cells, leaked mtDNA triggers NLRP3 and AIM2 inflammasomes assembly, inducing GSDM-dependent pyroptosis. 2、Up-regulating the expression of MRE11A in RA CD4+ T cells can reduce the generation of pyroptosis and slow down the progression of synovitis	(133, 137, 138)

Although pyroptosis is implicated in rheumatoid arthritis, the definite mechanism and pathway involved are not fully understood, and all these clinical drug targeting pyroptosis in RA require extensive preclinical studies. Just like the NLRP3 inhibitor MCC950 was stopped in clinical phase II due to its hepatotoxicity. Currently, there are few studies on targeted therapy drugs in pyroptosis, so we expect more studies to elaborate the molecular mechanism of PCD in RA, and we also expect that target therapy can open up a whole new way for the treatment of RA.

## Author contributions

DW and YL drafted the manuscript. DW, YL, and RX discussed and revised the manuscript. RX designed the research. These authors contributed equally: DW and YL. All authors contributed to the article and approved the submitted version.

## Glossary

**Table:**

RA	Rheumatoid arthritis
NSAIDs	non-steroidal anti-inflammatory drugs
DMARDs	disease modifying anti-rheumatic drugs
PCD	programmed cell death
caspase	cysteinyl aspartate specific proteinase
FLS	fibroblast-like synoviocytes
NLRP3	NOD-like receptor thermal protein domain associated protein 3
GSDM	gasdermin
NCCD	Nomenclature Committee on Cell Death
GSDMA	gasdermin A
GSDMB	gasdermin B
GSDMC	gasdermin C
GSDMD	gasdermin D
GSDME	gasdermin E
GSDM-CT	gasdermins C-terminal domain
GSDM-NT	gasdermins N-terminal domain
IL-18	Interleukin-18
IL-1β	Interleukin-1β
LDH	lactate dehydrogenase
LPS	lipopolysaccharide
PRR	pattern recognition receptor
CARD	caspase recruitment domain
PYD	pyrin domain
ASC	apoptosis-associated speck-like protein containing CARD
dsDNA	double-stranded DNA
NK cells	natural killer cells
ROS	reactive oxygen species
COX	cyclooxygenase
csDMARDs	conventional synthetic disease modifying anti-rheumatic drugs
bDMARDs	biological disease modifying anti-rheumatic drugs
tsDMARDs	target synthetic disease modifying anti-rheumatic drugs
MTX	methotrexate
SASP	sulfasalazine
LEF	leflunomide
NF-κB	nuclear factor-kappa B
JAK	Janus kinase
STAT	signal transducer and activator of transcription
MCP-1	monocyte chemoattractant protein-1
GM-CSF	granulocyte-macrophage colony stimulating factor
TLR4	Toll-like receptor 4
AIA-M	adjuvant-induced arthritis model
AA	adjuvant arthritis
MMPs	matrix metalloproteinases
PTX3	pentraxin 3
C1q	Complement component 1q
CIA	collagen-induced arthritis
POL β	polymerase β
PBMC	peripheral blood mononuclear cells
ASICs	Acid-sensitive ion channels
Pctx-1	Psalmotoxin-1
RANK	receptor activator of NF-kB
mtDNA	mitochondrial DNA
DMF	Dimethyl fumarate
NSA	necrosulfonamide
AIM2	Absent in melanoma 2
